# Myocardial Fibrosis, The Silent Instigator of Diastolic Dysfunction in Patients With Rheumatoid Arthritis

**DOI:** 10.33549/physiolres.935575

**Published:** 2025-06-01

**Authors:** Monika JALALI, Ján ŠTEVLÍK, Yashar JALALI, Andrea GAŽOVÁ, Ján KYSELOVIČ, Zdenko KILLINGER, Juraj PAYER

**Affiliations:** 1 Faculty of Medicine, Comenius University in Bratislava, and Fifth Department of Internal Medicine, University Hospital Bratislava, Bratislava, Slovak Republic; 2 Faculty of Medicine, Comenius University in Bratislava, Department of Pharmacology and Clinical Pharmacology, Bratislava, Slovak Republic

**Keywords:** Rheumatoid arthritis, Myocardial fibrosis, Diastolic dysfunction, Cardiovascular disease surveillance, Strain echocardiography

## Abstract

Rheumatoid arthritis (RA) is a chronic systemic inflammatory disease associated with increased cardiovascular morbidity and mortality. Myocardial fibrosis, a key pathological consequence of prolonged inflammation, contributes to diastolic dysfunction and the development of heart failure with preserved ejection fraction (HFpEF) in RA patients. Understanding its pathophysiology, early detection, and potential therapeutic strategies is crucial for improving patient outcomes. In this study we explore the underlying mechanisms of myocardial fibrosis in RA, focusing on immune-mediated pathways, oxidative stress, and extracellular matrix dysregulation, with concise look at the impact of immunosuppressive therapy on cardiac remodeling and role of speckle-tracking echocardiography (STE) in detecting subclinical myocardial fibrosis, emphasizing global longitudinal strain (GLS) as a promising surrogate marker.

## Introduction

Rheumatoid arthritis is a chronic inflammatory disease that primarily affects synovial joints, leading to progressive joint damage and functional impairment [1]. With a global prevalence of near 1 %, RA predominantly affects women, with peak onset occurring between 40 and 60 years of age. Beyond its joint-related manifestations, RA is a systemic disease with the potential to impact multiple organ systems. Extra-articular manifestations (ExRA) (include subcutaneous nodules, interstitial lung disease, vasculitis, and cardiovascular disease (CVD)), affect approximately 23 % of RA patients and increase mortality risk by threefold compared to those without ExRA [2]. Prolonged disease duration, advanced age, elevated inflammatory markers, and high levels of rheumatoid factor (RF) or anti-citrullinated protein antibodies (ACPA) have been shown to be linked with presence of ExRA.

Among the various ExRA, cardiovascular involvement represents a major contributor to morbidity and mortality in RA. Patients with RA have 10-fold higher risk for development of CVD in comparison to general population of same age under same risk factors [2]. Chronic systemic inflammation accelerates atherosclerosis, impairs endothelial function, and promotes vascular remodelling, increasing the risk of ischemic heart disease, heart failure, and stroke. Structural cardiac changes, such as increased left ventricular mass, myocardial fibrosis, and impaired diastolic function, are common in RA patients and are strongly associated with prolonged inflammation [2]. Myocardial fibrosis, a key pathological process in RA-related cardiac involvement, is closely linked to diastolic dysfunction. Chronic inflammation drives fibroblast activation and excessive extracellular matrix deposition, leading to myocardial stiffening, impaired ventricular relaxation, and elevated diastolic pressures [2]. This results in clinical symptoms such as fatigue and exertional dyspnoea manifesting as HFpEF. Moreover, fibrosis can disrupt myocardial architecture, altering ventricular elasticity and electrical conduction, further contributing to cardiac dysfunction in these patients.

Recent studies indicate that RA patients in both Europe and America exhibit a higher prevalence of diastolic dysfunction, with estimates suggesting that 20–30 % of RA patients experience some degree of this condition [3–5]. Among these individuals, a significant proportion progress to HFpEF [1]. Understanding the pathogenesis of myocardial fibrosis is therefore crucial for the primary management of these patients, as treatment options become severely limited once significant myocardial remodelling occurs and clinical symptoms of diastolic heart failure emerge, leading to a marked decline in quality of life [6]. Consequently, the primary strategy for these patients should prioritize prevention over treatment. Given this context, the impact of chronic inflammation on myocardial fibrosis and the potential role of immunosuppressive therapy in mitigating the inflammatory state warrant further investigation. Numerous studies have elucidated the intricate cascade of immune processes involved in myocardial fibrosis during RA progression, while others have examined the effects of immunosuppressive therapies on CVD risk in these patients. However, the debate remains unresolved regarding the balance between the potential benefits of short- and long-term immunosuppressive treatment and the associated cardiovascular risks or toxicities. This complexity underscores the need for further research, as a definitive answer to this question remains elusive.

In addition to the preventive management, continuous surveillance of CVDs, particularly the development of myocardial fibrosis, is of paramount importance. The detection of myocardial fibrosis has advanced significantly with the emergence of sophisticated imaging and diagnostic modalities in recent years. Cardiac Magnetic Resonance Imaging (CMR) is now widely regarded as the gold standard for detecting and quantifying myocardial fibrosis due to its superior ability to characterize myocardial tissue. Techniques such as late gadolinium enhancement (LGE) enable the identification of focal fibrosis by highlighting contrast retention in regions of increased extracellular matrix deposition. Moreover, T1 mapping and extracellular volume (ECV) quantification allow for the assessment of diffuse interstitial fibrosis, which is frequently observed in inflammatory diseases such as RA. These advanced techniques provide high-resolution images and quantitative data that are instrumental in evaluating the severity and distribution of fibrosis. However, CMR remains costly, has limited availability in many healthcare settings, requires prolonged scan times, and is contraindicated in certain patient populations, such as those with metallic implants or severe renal impairment due to the use of gadolinium-based contrast agents.

Echocardiography, however, remains a widely accessible and cost-effective tool for assessing cardiac function. While it does not directly detect myocardial fibrosis, advanced techniques such as STE and GLS analysis offer an indirect means of evaluating fibrotic changes by assessing myocardial deformation. The sensitivity and specificity of STE in detecting myocardial fibrosis have been extensively investigated in recent years, with promising results. However, the precise cutoff values for GLS in the detection of subclinical fibrosis and their predictive value for adverse cardiovascular events remain to be fully established.

In this review, we will comprehensively examine the latest insights into the pathophysiology of myocardial fibrosis in patients with RA, shedding light on the key mechanisms driving fibrotic remodelling and its clinical significance. Furthermore, we will explore the ongoing debate surrounding the impact of immunosuppressive therapy on RA-associated cardiovascular changes, evaluating both its potential benefits and concerns. Finally, we will discuss the emerging role of speckle-tracking echocardiography as a non-invasive tool for the surveillance of myocardial fibrosis, assessing its clinical utility in early detection and risk stratification.

## Pathophysiology of myocardial fibrosis in patients with rheumatoid arthritis

Myocardial fibrosis in RA arises as a consequence of a deeply intricate interplay between chronic inflammation, immune system disturbances, oxidative stress, and metabolic abnormalities, each contributing to the pathological remodelling of cardiac tissue [1,7]. At the core of this process lies the persistent systemic inflammation that defines RA, which exerts a profound influence on myocardial structure and function. Unlike transient inflammatory responses seen in acute conditions, the chronic inflammation in RA is sustained over years, if not decades, leading to continuous damage and remodelling within the heart [6]. This ongoing inflammatory environment fosters the activation of various cellular and molecular pathways that converge on the myocardium, ultimately driving fibrosis.

**Fig. 1:**
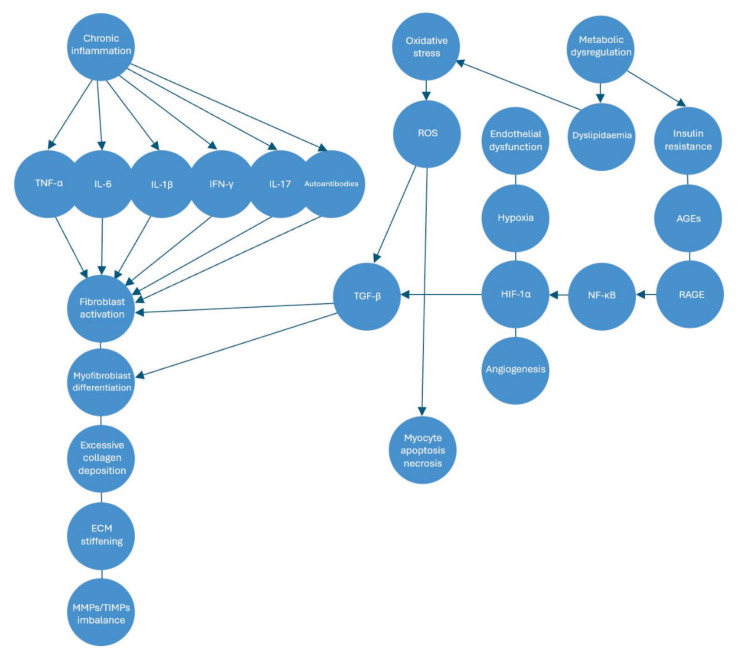
AGEs: Advanced Glycation End-products, ECM: Extracellular Matrix, HIF-1α: Hypoxia-Inducible Factor-1 alpha, IFN-γ: Interferon-gamma, IL: Interleukin, MMPs/TIMPs: Matrix Metalloproteinases/Tissue Inhibitors of Metalloproteinases, NF-κB: Nuclear Factor kappa B, RAGE: Receptor for Advanced Glycation End-products, ROS: Reactive Oxygen Species, TGF-β: Transforming Growth Factor-beta, TNF-α: Tumor Necrosis Factor-alpha.

A key element in this pathological transformation is the overproduction of pro-inflammatory cytokines, including tumor necrosis factor-alpha (TNF-α), interleukin-6 (IL-6), and interleukin-1 beta (IL-1β), which are persistently upregulated in RA (Fig. 1) [8]. These cytokines do not act in isolation but rather create an inflammatory milieu that alters the behavior of cardiac fibroblasts, the principal cells responsible for maintaining myocardial integrity [9]. In a healthy heart, fibroblasts play a fundamental role in extracellular matrix (ECM) homeostasis by regulating the delicate balance between synthesis and degradation of structural proteins [1]. However, under the prolonged influence of these cytokines, fibroblasts undergo pathological activation and transdifferentiate into myofibroblasts, an altered phenotype characterized by heightened proliferative capacity, increased contractility, and excessive ECM deposition [8]. Myofibroblasts acquire the ability to secrete large quantities of collagen, primarily types I and III, which are critical components of the myocardial ECM (Fig. 1) [6]. While collagen provides structural support necessary for the normal function of the heart, excessive accumulation disturbs the mechanical properties of the myocardium, leading to increased stiffness, reduced elasticity, and impaired cardiac compliance [2].

The dysregulation of ECM turnover further compounds the fibrotic process, as the delicate equilibrium between matrix metalloproteinases (MMPs) and their natural inhibitors, tissue inhibitors of metalloproteinases (TIMPs), becomes disrupted [8,10]. Under normal physiological conditions, MMPs play an essential role in ECM remodeling by breaking down excessive collagen and other ECM components, thereby preventing pathological accumulation [10]. However, in RA, this tightly regulated system is thrown into disarray. Chronic inflammation promotes an environment in which MMP activity is either excessively upregulated or suppressed inappropriately, leading to aberrant ECM deposition [10]. Simultaneously, TIMPs, which serve as endogenous inhibitors of MMPs, become dysregulated, further preventing appropriate ECM degradation and exacerbating fibrosis. This imbalance leads to a self-perpetuating cycle in which ECM components accumulate excessively, reinforcing the structural and functional changes in the myocardium [10].

The immune system is deeply implicated in the pathogenesis of myocardial fibrosis in RA, operating through a complex network of cellular and humoral responses that contribute to both the initiation and progression of fibrotic remodelling within the myocardium [11,12]. This immune-driven fibrosis is fueled primarily by the infiltration of activated CD4+ T-cells into the myocardial interstitium, where they orchestrate a cascade of inflammatory and fibrotic responses. Among the diverse CD4+ T-cell subsets involved, Th1 and Th17 cells are particularly pathogenic, exerting their effects through the secretion of pro-inflammatory cytokines that intensify the fibrotic process [11,12].

Interferon-gamma (IFN-γ), a signature cytokine of Th1 cells, plays a pivotal role in sustaining a pro-inflammatory microenvironment within the heart. IFN-γ stimulates macrophage activation, leading to the enhanced production of inflammatory mediators that further recruit and activate fibroblasts [12]. These activated fibroblasts, in turn, undergo phenotypic changes that drive excessive ECM deposition. Additionally, IFN-γ promotes antigen presentation by upregulating major histocompatibility complex (MHC) class II molecules on cardiac fibroblasts, thereby perpetuating immune activation and prolonging inflammatory damage. Alongside IFN-γ, interleukin-17 (IL-17), predominantly produced by Th17 cells, exacerbates myocardial injury by inducing the expression of pro-fibrotic genes in fibroblasts, stimulating neutrophil recruitment, and amplifying local inflammation [11]. IL-17 has also been shown to disrupt endothelial function, further contributing to myocardial fibrosis by impairing vascular integrity and promoting the infiltration of additional immune cells into cardiac tissue [11].

In parallel with T-cell-driven inflammation, B-cells contribute significantly to the immune-mediated fibrotic process through the production of autoantibodies. RA is characterized by the presence of RF and anti-citrullinated protein antibodies (ACPA), which not only serve as biomarkers of disease severity but also play direct pathogenic roles in myocardial fibrosis. These autoantibodies readily form immune complexes that deposit within the myocardium, triggering the activation of the complement cascade. Complement activation leads to a robust inflammatory response, marked by the recruitment of monocytes and neutrophils, the release of additional cytokines, and the generation of membrane attack complexes that inflict direct damage on cardiac cells. This localized immune-mediated injury accelerates the fibrotic process by further activating fibroblasts and stimulating excessive collagen synthesis. The deposition of immune complexes also contributes to microvascular dysfunction, which exacerbates tissue hypoxia and reinforces the conditions that drive fibrosis.

Beyond the direct effects of T-cells and B-cells, autoimmune mechanisms further sustain myocardial fibrosis through molecular mimicry, a process by which the immune system mistakenly targets myocardial antigens due to their structural resemblance to foreign antigens [13]. In RA, the chronic presentation of citrullinated proteins and other modified self-antigens fosters an environment in which the immune system becomes primed to attack host tissues, including the myocardium [13]. This pathological immune recognition leads to the continuous activation of autoreactive T-cells and B-cells, thereby perpetuating inflammation and fibrosis even in the absence of external inflammatory triggers [13]. Over time, this cycle of immune-mediated myocardial injury results in progressive structural remodeling, culminating in a fibrotic myocardium that is functionally impaired.

Oxidative stress plays a pivotal role in the progression of myocardial fibrosis in RA, acting as a critical link between chronic inflammation and pathological tissue remodeling (Fig. 1) [14]. The excessive production of reactive oxygen species (ROS), generated primarily by activated immune cells and dysfunctional mitochondria, creates a persistent state of oxidative damage within the myocardium [14]. This imbalance between ROS production and antioxidant defense mechanisms not only results in direct cellular injury but also amplifies the inflammatory and fibrotic processes that characterize RA-associated cardiac pathology [14]. ROS have a particularly detrimental impact on cardiomyocytes, promoting both apoptosis and necrosis, which in turn triggers fibroblast activation as part of a maladaptive repair response [14]. The ensuing myofibroblast differentiation and excessive ECM deposition further drive the stiffening of the myocardium, contributing to progressive functional impairment [14].

At the molecular level, oxidative stress serves as a potent activator of profibrotic signaling pathways, with transforming growth factor-beta (TGF-β) playing a central role in mediating these effects [14]. TGF-β is one of the most powerful drivers of fibrosis, orchestrating the conversion of fibroblasts into myofibroblasts, enhancing collagen synthesis, and suppressing ECM degradation (Fig. 1) [14]. The sustained activation of this pathway creates an environment in which fibrotic remodelling becomes self-perpetuating, reinforcing the pathological changes within the myocardium. Furthermore, oxidative stress exacerbates endothelial dysfunction, a well-recognized feature of RA, by impairing nitric oxide bioavailability [14]. This deficiency in nitric oxide, a key vasodilator and regulator of vascular homeostasis, contributes to vascular stiffening and reduces perfusion, fostering a hypoxic microenvironment [14]. Hypoxia further aggravates fibrosis by upregulating hypoxia-inducible factor-1 alpha (HIF-1α), a transcription factor that enhances TGF-β activity and promotes angiogenesis [14]. While angiogenesis is typically considered a reparative process, in the context of RA-related myocardial fibrosis, it paradoxically sustains inflammation and facilitates leukocyte infiltration, thereby prolonging the fibrotic response [14].

In addition to oxidative stress, metabolic disturbances associated with RA significantly contribute to the fibrotic process [15]. Dyslipidemia, commonly observed in RA patients, promotes lipid peroxidation, which generates toxic byproducts that further drive oxidative stress and inflammation (Fig. 1) [15]. These lipid-derived reactive species interact with cellular components, triggering signaling pathways that promote fibroblast activation and excessive ECM deposition [15]. Insulin resistance, another frequent metabolic abnormality in RA, compounds these effects by enhancing inflammatory and fibrotic responses through the activation of the receptor for advanced glycation end-products (RAGE) [15]. The accumulation of advanced glycation end-products (AGEs) within myocardial tissue serves as a chronic stimulus for nuclear factor kappa B (NF-κB) activation, a transcription factor that upregulates the expression of pro-inflammatory and profibrotic genes [15]. This metabolic-inflammatory axis creates a highly permissive environment for fibrosis, further amplifying the adverse cardiac consequences of RA [15].

Beyond these metabolic and inflammatory drivers, genetic and epigenetic factors also influence an individual’s susceptibility to myocardial fibrosis in RA [16]. Genetic predispositions, particularly polymorphisms in the HLA-DRB1 gene, have been linked to an increased risk of immune-mediated myocardial injury, highlighting the role of inherited immune dysregulation in the fibrotic process [16]. However, genetic factors alone are insufficient to explain the variability in disease manifestation, and epigenetic modifications have emerged as key regulators of fibrosis in RA [16]. Alterations such as DNA methylation and histone acetylation can lead to aberrant expression of inflammatory and fibrotic genes, reinforcing a persistent profibrotic state within cardiac fibroblasts and immune cells [16]. These modifications, influenced by chronic inflammation and environmental factors, may contribute to the irreversible nature of myocardial fibrosis, making early intervention crucial in preventing long-term cardiac complications [16].

The clinical consequences of myocardial fibrosis in RA are profound, as the progressive deposition of ECM components leads to increased myocardial stiffness, reduced compliance, and impaired ventricular relaxation. These changes primarily manifest as HFpEF, a condition characterized by diastolic dysfunction despite normal systolic performance. In the early stages, myocardial fibrosis remains subclinical, presenting a diagnostic challenge due to the absence of overt symptoms. As fibrosis progresses, the structural rigidity of the myocardium contributes to increased left ventricular filling pressures, impaired cardiac output, and eventually overt cardiovascular complications.

Beyond diastolic dysfunction and HFpEF, myocardial fibrosis in RA patients may also predispose to cardiac arrhythmias and sudden cardiac death (SCD). Fibrotic tissue alters the normal architecture and electrical conduction pathways within the myocardium, creating regions of slow conduction and electrical inhomogeneity, which serve as substrates for re-entrant arrhythmias [17]. Studies in patients with systemic inflammatory conditions have demonstrated a higher incidence of atrial fibrillation, ventricular arrhythmias, and QT interval prolongation, which may be potentiated by both systemic inflammation and fibrotic myocardial remodeling [18]. The risk of ventricular tachyarrhythmias is further amplified by cytokine-mediated cardiomyocyte injury and autonomic dysfunction frequently observed in RA [19]. Furthermore, myocardial fibrosis has been identified as a significant predictor of sudden cardiac death, especially in individuals with preserved ejection fraction, likely due to its arrhythmogenic potential and impairment of electromechanical coupling [20]. These findings underscore the importance of comprehensive cardiovascular surveillance in RA patients, not only for heart failure manifestations but also for electrophysiological complications that may carry grave prognostic implications.

The insidious nature of this process underscores the importance of early detection and targeted intervention, particularly strategies aimed at mitigating chronic inflammation, oxidative stress, and metabolic dysregulation. Without timely intervention, RA patients remain at heightened risk for progressive heart failure and related morbidity, emphasizing the urgent need for a multidisciplinary approach to cardiovascular risk management in this population.

## Does immunosuppressive treatment help to control myocardial fibrosis?

The implementation of immunosuppressive therapy is increasingly recognized for its potential cardioprotective effects, particularly in mitigating myocardial fibrosis and adverse cardiac remodelling through the suppression of immuno-inflammatory cascades. The theoretical foundation of this approach is grounded in the ability of immunosuppressants to inhibit key cytokines and signaling pathways (mentioned earlier) involved in fibrotic progression, thus preserving myocardial integrity. However, the extent of these benefits varies based on the specific therapeutic agents employed, patient comorbidities, and the complex interplay between immunomodulation and cardiovascular risks.

Corticosteroids, potent anti-inflammatory agents, exert their effects by inhibiting the release of pro-inflammatory cytokines such as TNF-α, IL-6, and IL-1β, reducing fibroblast activation and ECM deposition [21]. Furthermore, suppression of TGF-β, a critical mediator of fibrotic processes, reinforces their role in curbing myocardial fibrosis [22]. However, despite these advantages, chronic corticosteroid use is associated with significant cardiovascular detriments, including an increased risk of hypertension, insulin resistance, endothelial dysfunction, and oxidative stress, each of which can exacerbate ischemic injury and contribute to progressive cardiac dysfunction [23].

Methotrexate, a conventional synthetic disease modifying antirheumatic drug (csDMARD), has been widely studied for its cardioprotective potential in RA patients [24]. By inhibiting TNF-α and IL-6, methotrexate modulates fibroblast activation and limits ECM accumulation, thereby reducing myocardial fibrosis risk [24]. A systematic review by Roubille *et al*. demonstrated reduction in cardiovascular events among methotrexate users, alongside enhancement in endothelial function, highlighting its efficacy in reducing hypoxia-driven fibroblast activation [25]. However, methotrexate is not without its drawbacks, as it has been associated with pulmonary fibrosis and hepatotoxicity, which may indirectly exacerbate myocardial remodelling through systemic oxidative stress (Table 1) [26,27].

TNF-α inhibitors, widely utilized in RA management, effectively suppress inflammatory processes and slow disease progression. However, their influence on cardiac remodelling remains nuanced, with evidence suggesting negligible benefits in individuals below 50 years of age [28]. Emerging data indicate that older adults undergoing TNF-α inhibitor therapy may be at an increased risk of HF, necessitating further investigation into age-dependent cardiovascular implications (Table 1) [29]. Large-scale trials such as RECOVER and RENAISSANCE have failed to demonstrate any protective effect against HF, while the RENEWAL meta-analysis found no significant differences in mortality or hospitalization rates [30,31]. The ATTACH trial further underscored concerns regarding infliximab, which, at high doses, exacerbated HF symptoms and increased hospitalization rates, reinforcing the recommendation against TNF-α inhibitors in patients with severe decompensated HF [32].

On the other hand, IL-6 inhibitors, particularly tocilizumab, have demonstrated potential cardiovascular benefits [33]. Reports suggest that tocilizumab is associated with a lower incidence of major adverse cardiac events (MACE) compared to abatacept, with superior cardiovascular outcomes over TNF-α inhibitors, thus expanding the risk-benefit spectrum of biologic DMARDs (bDMARDs) in RA treatment (Table 1) [33].

The emergence of targeted synthetic DMARDs (tsDMARDs), particularly Janus kinase (JAK) inhibitors such as tofacitinib and baricitinib, represents a significant advancement in modulating intracellular pathways involved in myocardial fibrosis. These agents have demonstrated efficacy in reducing myocardial ECM accumulation and improving endothelial function (Table 1) [34,35]. However, their use is tempered by an increased thromboembolism risk, with studies indicating a twofold rise in thrombotic events among JAK inhibitor users compared to conventional DMARD recipients [36].

The intricate relationship between RA, CVD, and myocardial fibrosis underscores the necessity for precision medicine in therapeutic decision-making. While immunosuppressive therapies have shown notable cardioprotective effects, long-term safety considerations necessitate rigorous patient selection and continuous monitoring. Suppressing systemic inflammation in RA has been correlated with improved cardiovascular outcomes; however, the precise mechanisms linking cytokine inhibition to myocardial remodeling remain incompletely understood. Differentiating RA-related cardiac dysfunction from heart failure in the general population remains an essential focus for future research, particularly in optimizing treatment approaches for high-risk patient populations. In this sense the surveillance of RA patients specially for diagnosis of subclinical myocardial fibrosis in relation to disease activity and/or immunosuppressive treatment-effect gets even more clinical relevance. Moving forward, personalized therapeutic strategies that strike a balance between cardiovascular protection and fibrotic progression inhibition will be critical in refining disease management and improving patient outcomes.

## Does spackle tracking echocardiography detect myocardial fibrosis

Speckle-tracking echocardiography (STE), introduced in the late 1990s, revolutionized non-invasive myocardial mechanics assessment, with early 2000s research validating its feasibility and accuracy [39]. As echocardiography systems advanced with high-resolution automated software, STE gained widespread clinical and research acceptance by the 2010s, expanding its applications from ischemic heart disease and heart failure to myocardial fibrosis in systemic diseases like RA [39,40]. STE functions by tracking the displacement of speckles – natural acoustic markers within myocardial tissue – across sequential two-dimensional echocardiographic frames, enabling precise calculation of myocardial deformation (strain) and strain rate [41]. Longitudinal strain, a key STE parameter, measures myocardial deformation along the heart’s long axis, with negative values indicating contraction and positive values reflecting stretching [41]. Global longitudinal strain (GLS), the averaged strain across all left ventricular segments, provides a comprehensive assessment of left ventricular function. Since GLS detects subtle reductions in myocardial shortening before conventional echocardiographic abnormalities appear, it can serve as a valuable tool for early identification of myocardial fibrosis in systemic diseases [41].

Although clear cutoff values for GLS are not yet established, a GLS of −16 % or lower is generally considered abnormal. Most published studies have demonstrated a strong correlation between lower GLS values and adverse cardiovascular events, as well as cardiac remodeling. It appears that the lower the GLS value, the greater the degree of myocardial damage. In recent years several studies demonstrated the correlation of myocardial fibrosis detected through CMR with pathological values of GLS. In a study by Chadalavada *et al*. analyzing 41533 participants, authors found that increasing myocardial fibrosis (native T1) worsened GLS by 0.005 per 1 ms increase (*p*<0.001) [42]. Witt *et al*. demonstrated that heart failure patients exhibited significantly reduced GLS (−15.1 % vs. −19.3 % in healthy subjects), with worsening fibrosis on late gadolinium enhancement correlating with greater strain impairment (*p*<0.05) [43]. Similarly, Elkassas *et al*. in their recent publication indicated that hypertrophic cardiomyopathy (HCM) patients had significantly lower GLS (−12.5 % vs. −18.5 % in controls, *p*<0.001), with fibrosis-related segmental strain reductions preceding global dysfunction [44].

Several studies have further demonstrated the diagnostic performance of GLS in detecting myocardial fibrosis, revealing moderate to high sensitivity and specificity of this method. A study by Hu *et al*. assessing GLS in HCM patients, demonstrated that a GLS cutoff value of −16.5 % could predict significant myocardial fibrosis with 80.9 % sensitivity and 76.5 % specificity in correlation with CMR findings [45]. Similarly, in another recent study by Li *et al*., authors evaluated GLS predictive value for detection of myocardial fibrosis in HCM patients with preserved ejection fraction using contrast-enhanced CMR. They demonstrated that GLS was significantly reduced in patients with LGE, in association with myocardial fibrosis [46]. The study reported that diagnostic accuracy improved when GLS was combined with biomarkers such as N-terminal pro B-type natriuretic peptide (NT-proBNP) and left ventricular wall thickness, achieving a sensitivity of 70 % and specificity of 81.25 % [46].

The main challenge in using GLS as a surrogate marker for myocardial fibrosis, however, lies in its inherent variability due to sex differences, population heterogeneity, interobserver-inter vendor bias, and the presence of a diagnostic “grey zone”. Based on the latest EACVI recommendations, normal GLS values differ by sex, with males typically exhibiting values of −17 % and above, while in females, the threshold is −18 % and above [47].

Emerging evidence suggests that sex differences play a significant role in the pathophysiology and clinical manifestation of HFpEF, which is particularly relevant given the higher prevalence of both RA and HFpEF in women [48,49]. Women with RA are more prone to develop diastolic dysfunction and exhibit greater myocardial stiffness and impaired ventricular relaxation compared to their male counterparts, possibly due to sex hormone influences and intrinsic differences in myocardial remodeling pathways [49]. This might be the reason why GLS values also differ by sex, with healthy women (as mentioned above) typically presenting with more negative (i.e., better) GLS values than men [47].

Since, many studies establish pathological GLS cut-offs at −16 % or lower, this discrepancy creates a diagnostic grey zone (−16 % to −18 %) where the relationship between mildly reduced GLS and fibrosis remains uncertain. Furthermore, the challenge extends to asymptomatic middle-aged individuals (40s to 50s), where early fibrotic changes might be present but not yet functionally significant. Given the prognostic importance of subclinical fibrosis, particularly in populations at risk, defining clearer GLS thresholds and refining surveillance strategies are crucial for early detection and risk stratification specifically in patients with RA. Future research should focus on standardizing GLS measurement techniques across different imaging vendors, establishing sex-specific and disease-specific cut-off values, and integrating multimodal imaging approaches to enhance the diagnostic accuracy of myocardial fibrosis detection, ultimately improving early intervention strategies and patient outcomes.

## Conclusions

Myocardial fibrosis represents a critical cardiovascular complication in RA, progresing into diastolic dysfunction and HFpEF. Early detection and intervention are paramount, as advanced myocardial fibrosis significantly limits therapeutic options and negatively impacts quality of life. Although immunosuppressive therapy can plays a pivotal role in modulating “inflammatory mediated” myocardial fibrosis, a tailored, patient-specific approach is essential. Speckle-tracking echocardiography has emerged as a valuable non-invasive tool for detecting subclinical myocardial fibrosis, particularly through global longitudinal strain (GLS) assessment in recent years. Although GLS shows strong correlation with myocardial fibrosis detected by cardiac MRI, challenges remain regarding standardized cutoff values, sex-specific differences, and inter-vendor variability. Further research is needed to refine GLS thresholds and integrate multimodal imaging approaches to improve early diagnosis and risk stratification.

Overall, preventing myocardial fibrosis in RA requires a proactive strategy focused on reducing chronic inflammation, optimizing cardiovascular surveillance, and implementing individualized therapeutic approaches.

In addition to pharmacological strategies, lifestyle modifications play a pivotal role in the prevention and management of myocardial fibrosis in RA patients. Regular aerobic and resistance exercise has been shown to exert anti-inflammatory effects, improve endothelial function, and reduce arterial stiffness, thereby potentially attenuating fibrotic remodelling and enhancing overall cardiovascular resilience [50,51]. Moreover, structured exercise programs have demonstrated safety and efficacy in improving cardiorespiratory fitness, muscle strength, and functional capacity in RA patients, even in the presence of moderate joint involvement [52]. Dietary interventions, particularly those rich in anti-inflammatory and antioxidant nutrients – such as the Mediterranean diet – may also contribute to myocardial protection by reducing systemic inflammation, oxidative stress, and metabolic dysregulation [53]. Omega-3 fatty acids, polyphenols, and plant-based antioxidants have shown promise in modulating cytokine levels and improving lipid profiles, both of which are closely linked to fibrotic processes [54]. Incorporating these non-pharmacologic strategies into routine care not only complements immunosuppressive therapy but also addresses modifiable risk factors that influence the progression of cardiovascular disease in RA. A multidisciplinary approach that combines pharmacological, behavioral, and lifestyle-based interventions is therefore essential for optimizing long-term cardiovascular outcomes in this high-risk population.

Future studies should explore the long-term effects of complex immunosuppressive therapies on myocardial remodeling and further enhance the role of STE in clinical practice.

## Figures and Tables

**Table 1:** A concise summary of recently published data on the pros and cons of immunosuppressive treatment effects on cardiovascular disease in rheumatoid arthritis patients.

*Authors Study design*	Cohort	Medication	Anti-inflammatory effects related to CVDs	Adverse CV effect
*Fardet et al. [30] Retrospective*	341	Corticosteroids	Suppress TNF-α, IL-6, IL-1β; reduce fibroblast activation and ECM deposition; limit myocardial fibrosis.	Increased risk of hypertension, insulin resistance, oxidative stress, and endothelial dysfunction.

*Roubille et al. [19] Systematic review*	88255	Methotrexate	21 % reduction in cardiovascular events; reduces fibroblast activation and ECM accumulation; improves endothelial function.	Pulmonary fibrosis and hepatotoxicity, potential systemic oxidative stress worsening myocardial remodeling.
*Ridker et al. [31] Double-blinded RCT*	4786			Low-dose methotrexate is not effective at lowering blood levels of IL-1β, interleukin-6, or C-reactive protein, nor does it reduce cardiovascular events in patients with established coronary artery disease.

*Singh et al. [26] Systematic review*	14(study)	bDMARDs	Usage of Tocilizumab was associated with reduced risk of major cardiovascular event	
*Chung et al. [25] RCT*	150			Short-term TNFα antagonism with infliximab did not improve and high doses (10 mg/kg) adversely affected the clinical condition of patients with moderate-to-severe chronic heart failure.

*Hasni et al. [28] RCT*	30	tsDMARDs	Tofacitinib improves high-density lipoprotein cholesterol levels, cause improvements in arterial stiffness and endothelium-dependent vasorelaxation and decrease in type I IFN gene signature, and low-density granulocytes.	

bDMARDs: biologic Disease-Modifying Antirheumatic Drugs, CV: cardiovascular, CVD: cardiovascular disease, ECM: extracellular matrix, IFN: interferon, IL: interleukin, RCT: randomized control trial, TNF-α: tumor necrotic factor alpha, tsDMARDs: Targeted Synthetic Disease-Modifying Antirheumatic Drugs.
